# State-dependent modulation of low-threshold-current-regulated dendritic Ca^2+^ response in thalamic reticular neurons with extracellular electric fields

**DOI:** 10.1038/s41598-023-43611-y

**Published:** 2023-10-01

**Authors:** Yaqin Fan, Xile Wei, Meili Lu, Jiang Wang, Guosheng Yi

**Affiliations:** 1 Tianjin Key Laboratory of Process Measurement and Control, School of Electrical and Information Engineering, Tianjin University, Tianjin, China; 2 School of Information Technology Engineering, Tianjin University of Technology and Education, Tianjin, 300222 China

**Keywords:** Biophysics, Neuroscience

## Abstract

Deep brain stimulation (DBS) in thalamic reticular nucleus (TRN) neuron provides a novel treatment for drug-resistant epilepsy via the induced electrical field (EFs). However, the mechanisms underlying EF effects remain unclear. This paper investigated how EFs regulate low-threshold dendritic Ca^2+^ (dCa) response and thus contribute to the input–output relationship of TRN cell. Our results showed that EFs modulate firing modes differently in a neuronal state-dependent manner. At the depolarized state, EFs only regulate the spike timing of a somatic stimulus-evoked single action potential (AP) with less contribution in the regulation of dCa response but could induce the transition between a dendritic stimulus-evoked single AP and a tonic burst of APs via the moderate regulation of dCa response. At the hyperpolarized state, EFs have significant effects on the dCa response, which modulate the large dCa response-dependent burst discharge and even cause a transition from this type of burst discharge to a single AP with less dCa response. Moreover, EF effects on stimulation threshold of somatic spiking prominently depend on EF-regulated dCa responses and the onset time differences between the stimulus and EF give rise to the distinct effect in the EF regulation of dCa responses. Finally, the larger neuronal axial resistance tends to result in the dendritic stimulus-evoked dCa response independent of somatic state. Interestingly, in this case, the EF application could reproduce the similar somatic state-dependent dCa response to dendritic stimulus which occurs in the case of lower axial resistance. These results suggest that the influence of EF on neuronal activities depends on neuronal intrinsic properties, which provides insight into understanding how DBS in TRN neuron modulates epilepsy from the point of view of biophysics.

## Introduction

Acting as a neurological disorder characterized by spontaneous recurrent seizures, epilepsy brings trouble to many people’s life^1^. For epilepsy treatment, pharmacotherapy is currently the primary modality while there are still many patients whose seizures cannot be effectively controlled by drug. And surgical resection of the epileptogenic zone is only an option for a minority of patients^2^. Thus, deep brain stimulation (DBS) has drawn increased attention in treating medically intractable epilepsies, which is based on the modulation effect of electrical field (EF) on brain activities. And experiments pointed out that DBS could modulate the excitability of specific areas of the brain that participates in the initiation, propagation and maintenance of epileptic activity^3–6^. For example, the DBS in cerebellum^4^, thalamus^5^, hippocampus^6^ can delay or abolish the secondary generalization of seizures. However, there are no current studies that have favored one target over another.

Acting as the anatomical structure critical for arousal and EEG desynchronization, thalamic reticular nucleus (TRN) generates and modulates the occurrence of spike-wave discharges (SWDs) for absence seizures^7^, indicating that TRN can be a novel target to treat epilepsy with DBS. With Male Wistar rats, Nanobashvili et al.^8^ showed that the electrical stimulation of TRN suppressed the development of seizures. Also, Pantoja-Jiménez et al.^3^ found that the high-frequency stimulation of TRN could obtain an anti-epileptogenic effect and interrupt abnormal electroencephalogram recordings in rats. In their another study designed to determine appropriate stimulation parameters, they found that 10 min of preemptive high frequency stimulation in the TRN provoked a decrease in seizure severity compared to 60 min of preemptive stimulation or 10 min of responsive stimulation^9^. These studies reported the modulation effect of DBS in TRN on epilepsy with animal experiments. Differently, with simulations Wang et al.^10^ analyzed how DBS in TRN reduced the SWDs from the point of view of dynamic and pointed out that the suitable DBS at TRN reduces the SWD oscillation region by changing the GABA_B_-induced inhibition time delay. However, the biophysical mechanism about how DBS-induced EF influences the activities of TRN cell is still unclear.

Centrally located in the thalamocortical circuit, TRN receives monosynaptic glutamatergic inputs from the cerebral cortex and the thalamus and sends GABAergic projections to the thalamus^3, 11–13^. Actually, the magnitude of inhibition on thalamus relies critically on the firing mode of TRN neuron: tonic and burst^14, 15^. The tonic mode is associated with the processing of sensory information while the burst mode is related with the SWDs^3, 16^. Transitioning between tonic and burst mode relies on the presence of low-threshold dendritic Ca^2+^ current (*I*_T_) which is inactivated at relatively depolarized state and deinactivated at relatively hyperpolarized state^14^. The inactivation of *I*_T_ at depolarized membrane potential underlies the tonic single-spike activity while the deinactivation of *I*_T_ at hyperpolarized membrane potential serves to the generation of low-threshold spike (LTS) as a neuron receives sufficient excitatory inputs, which in turn facilitates the generation of burst discharge of Na^+^-dependent action potential (AP). With burst mode, the TRN neuron produces pronounced low-threshold dendritic Ca^2+^ (dCa) response at distal dendrites by activating significant Ca^2+^ current. These studies suggest that the voltage state plays an important role in determining the firing mode of TRN by modulating dCa response.

Decades of experimental and modeling studies have shown that the EF-induced polarization affects the spatial heterogeneity of membrane potential, which contributes to the variation of stimulation threshold for triggering action potential, spike timing and synaptic plasticity^17, 18^. Especially, our previous studies pointed out that the EF-induced dendritic polarization plays an important role in the neuronal input–output relationship by affecting dendritic activities^19–22^. Therefore, the change of dendritic transmembrane potential caused by EFs seems to regulate the input–output function of TRN neuron via modulating *I*_T_-dependent dCa response. Moreover, we found that the onset time difference between the stimulus and EF plays an important role in determining the threshold for triggering dendritic Ca^2+^ spike owning to the state-dependent of Ca^2+^ channel dynamic^22^. And the axial resistance influences the spatial heterogeneity of membrane potential by determining the connectivity between compartments. Hence, we hypothesized that EF could affect the input–output relationship of TRN neuron by modulating dCa response, which depends on the voltage state, input timing and axial resistance.

To verify our hypothesis, we adopted a computational model of TRN cell receiving somatic and dendritic inputs to produce tonic and burst firing mode. Then we introduced EF stimulation into TRN cell with the extracellular mechanism of NEURON and characterized how the applied EFs modulate the firing mode and the stimulation threshold for triggering AP by affecting dCa response. We found that the change of firing mode in response to EF stimulation not only relies on the input location but also depends on the voltage state. At relatively depolarized state, EFs only regulate the spike timing of a somatic stimulus-evoked single AP with less contribution in the regulation of dCa response, which is consistent with the influence of somatic polarization on spike timing^23^. But for dendritic stimulus, it could induce the transition between a single AP and a tonic burst of APs via the moderate regulation of dCa response. At relatively hyperpolarized state, both for dendritic and somatic inputs EF-regulated dCa response has significant variation, which modulates large dCa response-dependent burst discharge. Unlike the approximately linear continuous regulation of dCa response to a dendritic stimulus, EF variations cause a steep change between the subthreshold and suprathreshold dCa responses to a somatic stimulus, which leads to the transition from a large dCa response-dependent burst discharge to a single AP with less dCa response. Further, our results showed that the modulatory effects of EF on stimulation threshold for triggering somatic AP prominently depend on the onset time differences between the stimulus and EF, since the onset time differences give rise to distinct effect in the EF regulation of dCa responses. We also found that the increasing axial resistance tends to introduce space heterogeneous distribution of neuronal state along neuronal axis, which results in the pronounced dendritic stimulus-evoked dCa response independent of somatic state. This simulation result is consistent with the experimental results reported by Crandall et al.^14^, which suggest that the axial resistance is a key factor in determining the state-independent dCa response. Especially, with larger axial resistance the EF could reproduce the similar somatic state-dependent dCa response to dendritic stimulus which occurs in the case of lower axial resistance. These findings suggest that the modulatory effect of EF on neuronal activities is dependent on neuronal intrinsic properties, which provides insight into understanding how DBS in TRN suppresses epilepsy from the point of view of biophysics.

## Results

### The modulation effect of EF on neuronal output depends on voltage state

**Figure 1:**
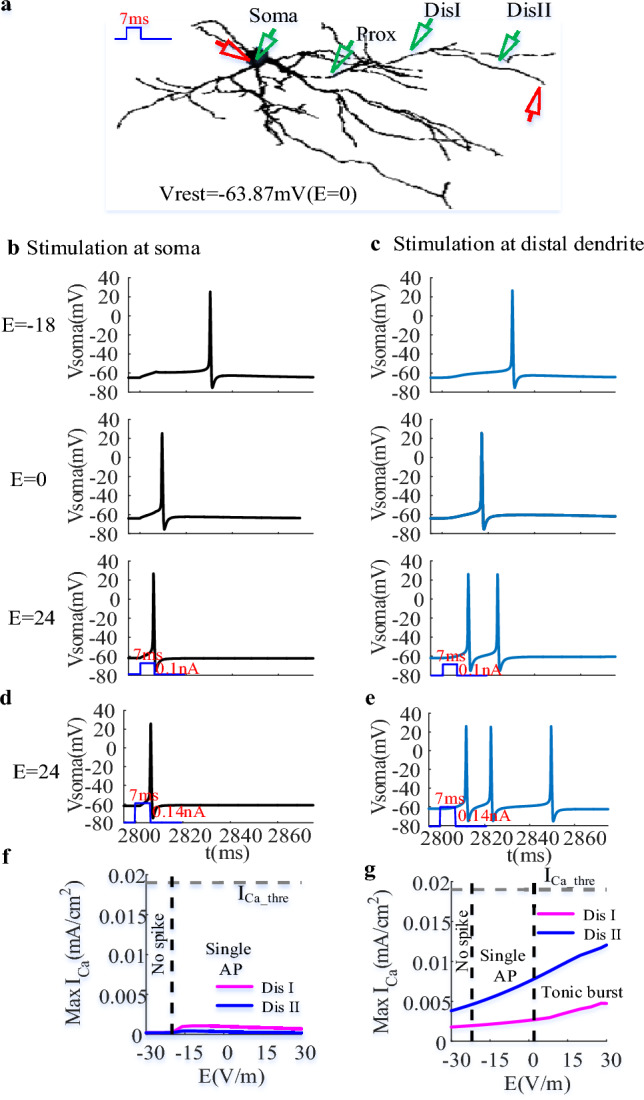
EF modulates the neuronal output of TRN neuron at relatively depolarized state. (**a**) the schematic diagram of a TRN cell to observe the change of neuronal output in response to somatic or dendritic current injection (7 ms; red triangles) as the initial membrane potential of soma was held at − 63.87 mV. The green triangles represented the recording sites at soma, proximal and distal dendrites. (**b**) and (**c**) The varying somatic membrane potential with time at E = − 18 V/m, E = 0 (control case), E = 24 V/m as the somatic and dendritic current injection was 0.1nA. (**d**) and (**e**) The change of somatic membrane potential with time at E = 24 V/m as the amplitude of somatic and dendritic current pulse was 0.145nA. (**f**) and (**g**) The Ca^2+^ response at medial (magenta) and distal (blue) dendrites as the TRN neuron received somatic and dendritic current stimulation respectively. The grey dotted line represented the threshold for triggering large dCa response. The black dotted lines represented the critical state for neuronal output.

**Figure 2:**
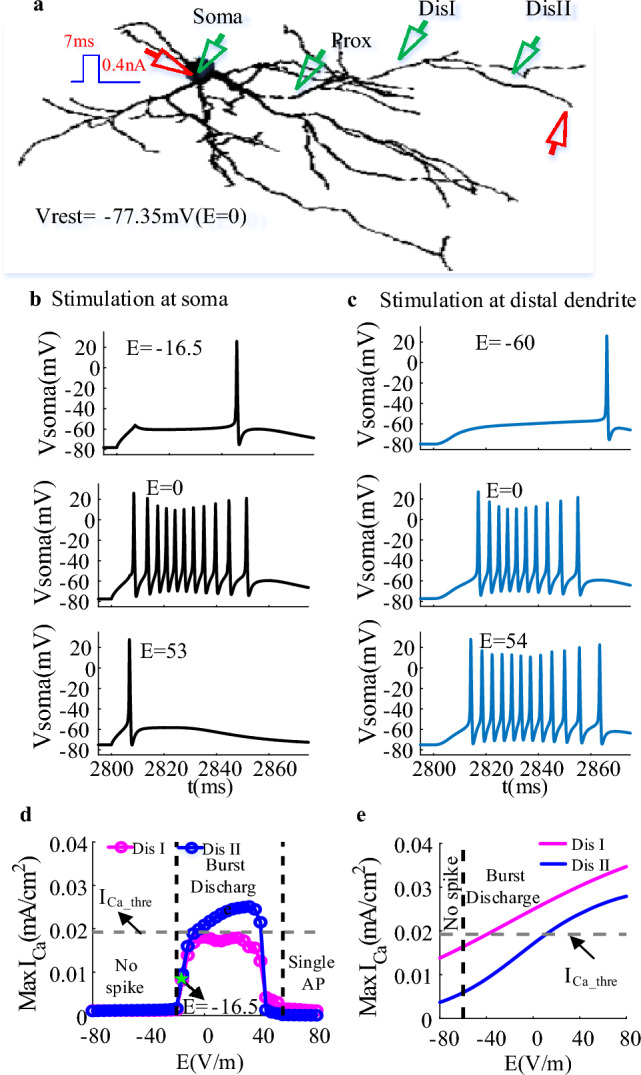
EF modulates the neuronal output of TRN neuron at relatively hyperpolarized state. (**a**) the schematic diagram of a TRN neuron receiving somatic or dendritic current injection (7 ms;0.4nA, red triangle) as the initial membrane potential of soma was held at − 77.35 mV. The green triangles at soma, proximal and distal dendrites represented the recording electrode. (**b**) The change of somatic membrane potential with time as the TRN neuron received somatic current injection at E = − 16.5 V/m, 0, 53 V/m. (**c**) The variation of somatic membrane potential with time as the TRN neuron received dendritic current injection at E = − 60 V/m, 0, 54 V/m. (**d**) and (**e**) The Ca^2+^ response at medial (magenta) and distal (blue) dendrites for somatic (marked with circle) and dendritic current stimulation respectively. The grey dotted line represents the threshold for triggering large dCa response. The black dotted lines represent the critical state for neuronal output. And the green pentagram represents E = − 16.5 V/m.

As described in Introduction, the voltage state plays an important role in determining the inactivation or deinactivation of *I*_T_, influencing the appearance of dCa response, which in turn affects the transition between tonic and burst mode. In this section, we characterized how voltage states contribute to the modulatory effect of EF on firing mode in response to a brief somatic or dendritic current by holding the initial somatic membrane potential at relatively depolarized and hyperpolarized state, as shown in Figs. 1 and 2.

We found that both somatic and dendritic inputs could elicit a single AP in control case as the initial membrane potential of soma is held at relatively depolarized state (Fig. 1b,c). Unlike the weak dCa response evoked by somatic stimulus, there exists more pronounced dCa response for dendritic inputs. As the TRN neuron is subjected to EF stimulation, the variation of somatic stimulus-evoked dCa is weak (Fig. 1f). Thus, we observed a change of spike timing of single AP (Fig. 1b,d). This result is similar to the experimental phenomenon that reported the variation of neuronal spike timing with somatic polarization^23^. While for dendritic inputs the anodal EF stimulation results in a moderate increase of dCa response via the dendritic hyperpolarization (Fig. 1g), giving rise to a transition between a single AP and a tonic burst of APs (Fig. 1c,e). Especially, the number of AP in response to a brief dendritic current is same as the tonic burst evoked by a periodic stimulus as the amplitude of the brief current injection increases (Fig. 1e), indicating that EF-induced hyperpolarization at distal dendrites plays a crucial role in modulating the neuronal output by affecting dCa response.

As the initial somatic membrane potential is held at relatively hyperpolarized state, both the somatic and dendritic inputs result in pronounced dCa response by activating* I*_T_, which in turn leads to the generation of burst discharge (Fig. 2b,c). In this case, the dendritic stimulus-evoked dCa response increases linearly with EF intensity, which results in the neuronal output transition from no spike to burst discharge (Fig. 2d,e). Differently, for somatic inputs there appears a steep change between the subthreshold and suprathreshold dCa responses in response to EF stimulation. In this case, the firing mode transitions between a large dCa response-dependent burst discharge to a single AP with less dCa response (Fig. 2b,c).

### EF-regulated stimulation threshold depends on the input timing

In our previous study, we found that the time interval between dendritic inputs and the onset of EF plays an important role in determining the dynamic of Ca^2+^ channel, which leads to the state-dependent modulation effect of EF on the threshold for triggering dendritic Ca^2+^ spike^22^. Similarly, here we determined the threshold for triggering action potentials at relatively hyperpolarized state when the time interval between somatic input *I*_soma_ or dendritic input *I*_dend_ and the onset of EF was 2 ms (i.e., transient state) and 400 ms (i.e., steady state).

**Figure 3:**
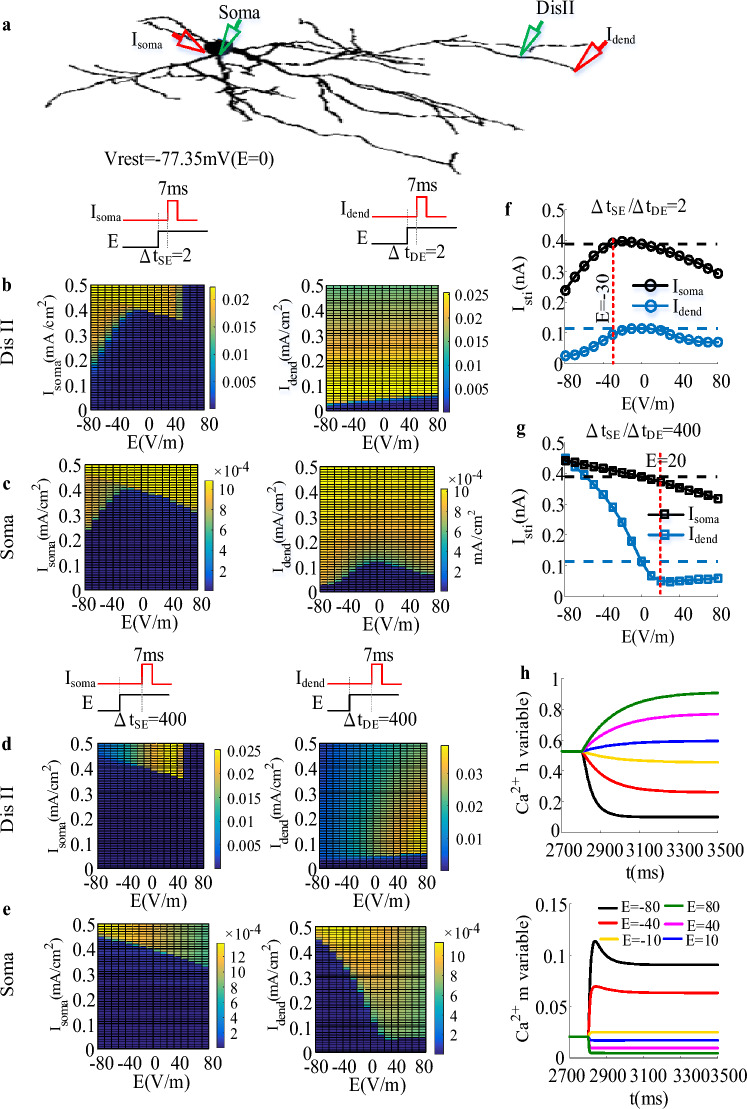
The modulation effect of EF on stimulation threshold *I*_dend_ and *I*_soma_ for triggering action potential as the initial membrane potential of soma was held at − 77.35 mV. (**a**)The schematic diagram of a TRN neuron receiving somatic or dendritic current injection (7 ms, red triangle) as the initial membrane potential of soma was held at − 77.35 mV. The green triangles at soma and distal dendrites represent the recording electrode. (**b**) and (**c**) The variation of peak Ca^2+^ response at distal dendrites (**b**) and soma (**c**) against EF intensity for $\Delta t_{SE} /\Delta t_{DE} = 2ms$ as the somatic(left) and dendritic (right) input increases from 0 to 0.5nA at step of 0.01nA. (**d**) and (**e**) similar to (**b**) and (**c**) but for $\Delta t_{SE} /\Delta t_{DE} = 400ms$. (**f**) and (**g**) The threshold *I*_dend_ (blue) and *I*_soma_ (black) for triggering action potential plotted as a function of EF for $\Delta t_{SE} /\Delta t_{DE} = 2ms$ (**f**) and $\Delta t_{SE} /\Delta t_{DE} = 400ms$(**g**). The black and blue dotted line respectively indicates the somatic and dendritic threshold at E = 0 V/m. (**h**) Ca^2+^ gating variable *h* and *m* at E = − 80 V/m (black), E = − 40 V/m (red), E = − 10 V/m (yellow), E = 10 V/m (blue), E = 40 V/m (magenta), and E = 80 V/m (green).

As shown in Fig. 3, the time interval between *I*_soma_ and the onset of EF is referred to $\Delta t_{SE}$ and the time interval between *I*_dend_ and the onset of EF is referred to $\Delta t_{DE}$.We found that in control case the threshold *I*_dend_ for triggering action potentials is smaller than *I*_soma_(Fig. 3f,g). As the TRN neuron is exposed to EF stimulation, both threshold *I*_dend_ and *I*_soma_ for triggering action potentials show distinct evolutions with the intensity of EF in two states. When the neuronal input and the onset of EF coincide within a few milliseconds (i.e., $\Delta t_{SE} /\Delta t_{DE} = 2ms$), *I*_dend_ and *I*_soma_ first increase and then decrease (Fig. 3f). In this case, for *I*_dend_ both anodal and cathodal stimulation facilitate the initiation of action potential. Differently, for *I*_soma_ the strong cathodal stimulation (E < − 30 V/m) and anodal stimulation facilitate the generation of action potential. With − 30 V/m < E ≤ 0, it is inhibited to trigger action potential by the applied fields. After field-induced polarization reaches a steady state, *I*_soma_ decreases as a function of EF (Fig. 3g) while *I*_dend_ first decreases within − 80 V/m < E ≤ 20 V/m and then slowly increases when the intensity of EF is above 20 V/m. In this case, the cathodal stimulation inhibits the generation of action potentials while anodal stimulation facilitates the initiation of action potential.

As described above, both the somatic and dendritic inputs can trigger dCa response by activating *I*_T_ as the initial membrane potential of soma is held at relatively hyperpolarized state. Under cathodal stimulation, the distal dendrites are depolarized while the soma is hyperpolarized. At the onset of cathodal stimulation, there is no change in the gating variable of *I*_T_ (Fig. 3h), the EF-induced depolarization facilitates the initiation of dCa response (Fig. 3b). Compared with the somatic hyperpolarization, the amplifying effect of dCa response on afferent inputs plays a dominant role in determining neuronal output, promoting the generation of action potential (Fig. 3f). On the contrary, in case of anodal stimulation the distal dendrites were hyperpolarized while the soma was depolarized. When the onset of EF and somatic or dendritic input are timed, there is no change in the channel inactivation of *I*_T_ (Fig. 3h). Although the EF-induced hyperpolarization increases the threshold at dendrites for triggering dCa response, however, there still exists dCa response at distal dendrites to amplify the dendritic inputs. Under this condition, compared with dendritic hyperpolarization somatic depolarization plays a dominant role in determining the decrease of the threshold *I*_dend_ for triggering action potential (Fig. 3f). Unlike the transient state, sustained field-hyperpolarization increases the Ca^2+^ gating variable *h* and channel availability (Fig. 3h), which results in more pronounced dCa response (Fig. 3c). In this case, the combination of somatic depolarization and more pronounce dCa response has a synergetic effect on decreasing the threshold *I*_dend_ for trigger action potential. With E > 20 V/m, the strong dendritic hyperpolarization decreases significantly Ca^2+^ gating variable *m* and the modulation effect of EF becomes saturation for stronger EF intensity. Therefore, the threshold *I*_dend_ for triggering action potential increases slowly. In case of the threshold *I*_soma_ for triggering action potential, since the dCa response is evoked by backpropagation action potential, the threshold for triggering action potential is dominant determined by the somatic depolarization (Fig. 3d), which results in a decreased *I*_soma_. The sustained field-induced depolarization decreases Ca^2+^ gating variable *h* (Fig. 3h) and leads to the inactivation of *I*_T_. This decreases the channel availability of *I*_T_, resulting in the failure to evoke dCa response. Thus, the combination of somatic hyperpolarization and the disappearance of dCa response results in the increase of *I*_soma_ and *I*_dend_ as $\Delta t_{SE} /\Delta t_{DE}$ extends.

### Axial resistance contributes to the modulatory effect of EF on spatial heterogeneity

**Figure 4:**
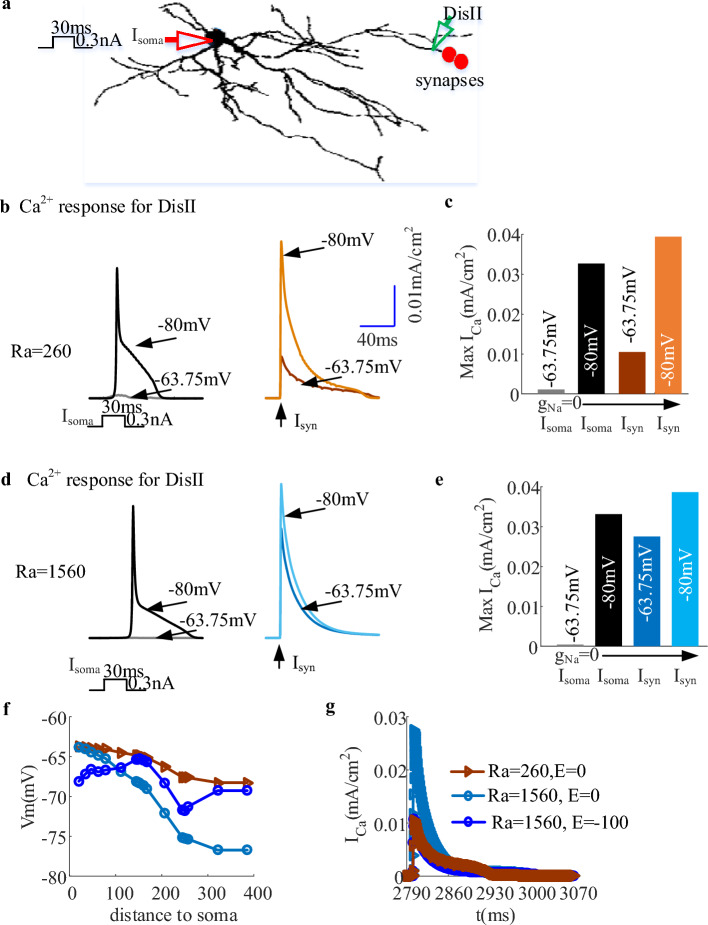
The influence of varying axial resistance (Ra) on dCa response under EF stimulation. (**a**) The schematic diagram of observing the dCa response at distal dendrites (green triangle) as the TRN neuron receive dendritic synaptic inputs (red dot) or somatic current injection (red triangle) in case of Ra = 260 $\Omega \;cm$ and Ra = 1560 $\Omega \;cm$. (**b**) The Ca^2+^ response measured at distal dendrite in the presence of Na^+^ blocker resulting from somatic current injection (*I*_soma_) and synaptic inputs at distal dendrites (*I*_syn_) in case of Ra = 260 $\Omega \;cm$ by holding the somatic membrane potential at either − 80 mV (black for *I*_soma;_ Orange for *I*_syn_) or − 60 mV (grey for *I*_soma;_ brown for *I*_syn_). (**c**)The plot shows the amplitude of the synapse-evoked responses in these conditions relative to the Ca^2+^ response evoked by somatic current injection as the soma was held at − 80 or − 63.75 mV. (**d**) similar to (**b**) but for Ra = 1560 $\Omega \cdot cm$. Here the dark and light blue represent the synapse-evoked dCa as the soma was held at − 63.75 mV or − 80 mV, respectively. (**e**) similar to (**c**) but for Ra = 1560 $\Omega \cdot cm$. (**f**) and (**g**) The modulation effect of EF on membrane potential (**f**) along dendrites from soma and the dCa response (**g**) for different Ra as the initial membrane potential of soma was held at − 63.75 mV. Ra = 260 $\Omega \;cm$, E = 0 and Ra = 1560 $\Omega \;cm$, E = 0 represents the case that Ra = 260 $\Omega \;cm$ or Ra = 1560 $\Omega \;cm$ without EF stimulation. Ra = 1560 $\Omega \;cm$, E = − 100 V/m represents the case that the TRN neuron with Ra = 1560 was under cathodal EF stimulation. Here, for distal dendrites *g*_T_ = 0.8 mS/cm^2^.

Decades of experimental and modeling studies pointed out that EF regulates neuronal activities by resetting the resting potential which affects neuronal spatial heterogeneity1^7, 19^. Acting as an important passive parameter, axial resistance (Ra) plays a crucial role in determining the space heterogeneous distribution of neuronal membrane potential. In vivo, the TRN neuron receives synaptic inputs at distal dendrites instead of current injection. To prove whether Ra contributes to the influence of EF on neuronal activities by affecting spatial heterogeneity, in this section we studied the variation of EF-regulated dCa response in response to synaptic inputs at distal dendrites for different Ra, as shown in Fig. 4.

With Ra = 260 $\Omega \;cm$, the dCa response in case of − 80 mV is much more pronounced than that at − 63.75 mV. However, with Ra = 1560 $\Omega \;cm$ the TRN cell can produce a robust dCa response no matter what kind of neuronal state (Fig. 4b), which is consistent with the experimental finding of Crandall et al.^14^ and reveals the important role of pronounced space heterogeneous distribution of   neuron along somatic-dendritic axis in determining state-independent dCa response. It not difficult to know that the space heterogeneity of membrane potential was changed by the applied EF as the TRN cell is subjected to EF stimulation. Therefore, we tested whether the EF-regulated space heterogeneity could transition the dendritic activities with different Ra, as shown in Fig. 4f,g. It can be found that the cathodal EF-induced depolarization for Ra = 1560 $\Omega \;cm$ can reproduce the resting potential of distal dendrites in case of Ra = 260 $\Omega \;cm$. Thus, under the EF modulation the TRN neuron with larger Ra can reproduce the similar somatic state-dependent dCa response to dendritic synapses which occurs in the case of lower axial resistance. It is pointed out that the dendritic morphology can undergo significant changes in epilepsy^37^. Considering the important role of axial resistance in dendritic morphology, our results suggest that DBS is capable to treat epilepsy caused by dendritic morphology by modulating the membrane potential heterogeneity through EF-induced polarization.

## Discussion

TRN acts as an important structure between the thalamus and the cortex, it receives collaterals from corticothalamic and thalamocortical neurons and exerts powerful inhibition on these neurons. In recent years, TRN has gained increased attention due to the important role in the development of epilepsy^24, 25^. Especially, several reviews pointed out the potential of TRN in the treatment of drug-resistant epilepsy^26^, which suggests that TRN may be a potential target for DBS. Therefore, many studies explored the modulation effect of DBS in TRN on suppressing seizures with animal experiments and simulation^3, 10^. However, few study discovered the biophysical mechanism about how the DBS-induced EFs modulates the input–output relationship of TRN neuron. In our study, we found that at relatively depolarized state the EF-induced polarization only changes the spike timing of single AP in response to somatic inputs due to the less contribution to the regulation of dCa response. While for a brief dendritic pulse, the dendritic hyperpolarization results in the transition between a single AP and a tonic burst of APs via increasing dCa response. At relatively hyperpolarized state, in control case both dendritic and somatic inputs can trigger   burst discharge by evoking significant dCa response. Under this condition, for somatic input the weak dendritic hyperpolarization facilitates the generation of burst discharge by increasing dCa response while the strong dendritic hyperpolarization promotes the generation of single AP by inhibiting the initiation of dCa response. Unlike the steep change of dCa response evoked by somatic inputs, the dCa response increases linearly with EF intensity, therefore cathodal stimulation inhibits burst discharge while anodal stimulation facilitates the generation of burst discharge. These findings indicate that the voltage state plays an important role in the modulatory effect of EF on neuronal output by affecting the dCa response, depending on the activation site of synaptic inputs.

Recently, the contribution of EF-induced dendritic polarization to the alternation of stimulation threshold for triggering Na^+^ based AP has been measured in some pyramidal cells using electrophysiological techniques and modeling studies^19, 20, 27^. Here we studied how EF-regulated dendritic Ca^2+^ response contributes to the EF-regulated stimulation threshold for triggering AP as the soma was held at relatively hyperpolarized states. We found that the effects of EF on stimulation threshold for triggering AP depends on the timing of synaptic inputs. When the somatic and dendritic inputs coincide with the onset of EFs within a time window of several milliseconds, EF-induced depolarization at dendrites under cathodal stimulation decreases the stimulation threshold of triggering AP by facilitating the initiation of dCa response while in case of anodal stimulation somatic depolarization decreases the stimulation threshold even if the dendritic hyperpolarization suppresses the initiation of dCa response. Thus, the somatic polarization and dCa response regulated by dendritic polarization have an opposite effect on decreasing the threshold for triggering action potential. Differently, the sustained EF-induced dendritic depolarization inhibits the initiation of dCa response by inactivating Ca^2+^ channels, which inhibits the initiation of AP as it is combined with somatic hyperpolarization. And the sustained EF-induced hyperpolarization leads to more pronounced dCa response by de-inactivating Ca^2+^ channels, facilitating the generation of AP when it is combined with somatic depolarization. In this case, the somatic polarization and dCa response regulated by dendritic polarization have a synergetic effect on decreasing or increasing the threshold for triggering AP, depending on the EF polarity.

Decades of experimental and modeling studies have paid more attention to the modulatory effect of EF on pyramidal neuron, since its asymmetric dendritic morphology of pyramidal neurons results in significant polarization^17, 22, 27–33^. However, few attentions were paid to the modulation effect of EF on interneurons due to its weak somatic polarization. In our study, we found that the applied EF at TRN cell induces significant dendritic polarization (Fig. 1B). In our previous studies^19, 22^ we found that dendritic polarization could modulates the dendritic integration, which further affects neuronal input–output relationship. In this paper, we found that EF effects on stimulation threshold of somatic spiking prominently depend on EF-regulated dCa responses and the onset time differences between the stimulus and EF give rise to the distinct effect in the EF regulation of dCa responses, indicating a state-dependent modulation effect of EF on stimulation threshold for triggering AP. It has been indicated that the dendritic *I*_T_ plays an important role in determining the firing mode of TRN neuron^14^. In this sense, our results suggest that *I*_T_-dependent dCa response may be one potential mechanism through which the DBS-induced EF affects interneurons. We also found that the EF direction with respect to the cell preferentially governs the EF-regulated stimulation threshold for triggering action potential, since it determines whether the dCa response is facilitated or suppressed. Earlier studies^17, 18, 22^ suggest that the EF orientation with respect to somato-dendritic axis plays a crucial role in the modulation effect of EF on neuronal activities, which is comparable to our prediction.

It is pointed out that the effects of ANT (anterior thalamic nucleus)-DBS on modulating epilepsy exhibit highly individual variability among subjects, which are difficult to predict and interpret. Such variability may result from the interaction of many factors, including the stimulatory dose, the neuronal morphology and the biophysics of specific cellular components and so on^22^. Our finding also indicates that the influence of EF on the input–output function of TRN cell is highly variable. Two factors are identified in our simulations. One is the intensity and polarity of EF, which directly influences the dCa response by modulating the dendritic polarization. The other factor is neuronal biophysics, including voltage state and the timing and location of synaptic inputs, which significantly affects the magnitude of dCa response. The variability in the effects of EF on dCa response will be translated into distinct regulations of neuronal computation and network dynamics^22^. Thus, simulating how EF-regulated dendritic Ca^2+^ response contributes to the input–output function of TRN cell under EF stimulation is essential for us to understand the subject-specific modulations of epilepsy with DBS.

There are several limitations of technical consideration in our research. First, we only used one kind of TRN neuron to explore the modulation effect of EF. However, the modulatory effect of EF relates to the neuronal morphology^18, 33^. Second, there are multiple voltage-gated channels in dendrites, including hyperpolarization-activated cation channels, which are shown to contribute to the variation of dendritic polarization^20^. And our multi-compartmental model didn’t include complex voltage-dependent channels in order to obtain the modulation effect of EF on firing mode transition easily. Third, the afferent axons and presynaptic terminals, whose dynamics influences the release of glutamate transmitter^32^, weren’t defined in our stimulations. We reproduced the synaptic inputs with a double exponential function. These factors also played an important role in EF modulation. However, these limitations don’t influence the effectiveness of our results, which provides insight into the mechanism about how EF interacts with cellular responses.

### Limitations

It should be noted that the DBS mechanism of action is too complex to be summarized from the single neuron level. And it is more feasible that the microscale perspective reflects the action of a mesoscale (network of multiple neurons in single or adjacent nuclei) and macroscale ones (network of distant nuclei and cortical areas liked by structural and functional connections). In our studies, we pointed out the mechanism about how DBS-induced EF affects the firing mode and stimulation threshold of TRN cell by modulating dCa response. Thus, we hope that our computational results can provide a theoretical guidance for understanding the DBS-modulated epilepsy.

## Conclusion

Here we studied how the variation of dCa response under DBS-induced EF stimulation contributes to the change of firing mode and stimulation threshold of TRN neuron. We found that the modulation influence of EF on firing mode depends on the neuronal state. At the depolarized state, EFs only regulate the spike timing of a somatic stimulus-evoked single action potential (AP) with less contribution in the regulation of dCa response but could induce the transition between a dendritic stimulus-evoked single AP and a tonic burst of APs via the moderate regulation of dCa response. At the hyperpolarized state, EFs have significant effects on the dCa response, which modulate the large dCa response-dependent burst discharge and even cause a transition from this type of burst discharge to a single AP with less dCa response. And the stimulation threshold of somatic spiking is affected significantly by the onset time differences between the stimulus and EF due to the distinct modulation effect of EF on dCa responses. Further, we found that the larger neuronal axial resistance tends to result in the dendritic stimulus-evoked dCa response independent of somatic state. In this case, the EF application could reproduce the similar somatic state-dependent dCa response to dendritic stimulus which occurs in the case of lower axial resistance. These results are helpful to understand the interactions between applied fields and TRN cell’s response from the point of view of biophysics.

## Methods

We used a biophysical model of a TRN neuron to quantify the influence of EF on dendritic *I*_T_, which is taken from previous work^34^. This model included a cell body, 20 proximal dendrites and 60 distal dendrites. Its membrane resistance, axial resistance and membrane capacity were $R_{m} = 20\;K\Omega \;cm^{2}$, $R_{{\text{a}}} = 260\;\Omega \;cm$, and $C_{m} = 1\;\mu F\;cm^{ - 2}$, respectively.

Its voltage-dependent conductances were modeled using a Hodgkin-Huxley type of kinetic model. To produce APs, fast sodium (*I*_Na_) and potassium (*I*_K_) current were introduced in the soma, and their kinetics were followed^24^, which are calculated using the general form of

1 $$I_{{{\text{ion}}}} = g_{ion} m^{a} h(V_{m} - E_{ion} )$$

Here *E*_ion_ was the reversal potential for individual channel, which was *E*_Na_ = 50 mV, *E*_K_ = − 100 mV, and *E*_L_ = − 82 mV. *g*_ion_ was the local conductance density. In the soma, *g*_Na_ = 100 mS/cm^2^, *g*_K_ = 80 S/cm^2^.* m* was an activation gating variable with *a* order kinetics, and *h* was an inactivation gating variable. The time and voltage dependency of each gating variable (*x*) was given by

2 $${{dx} \mathord{\left/ {\vphantom {{dx} {dt}}} \right. \kern-0pt} {dt}} = \alpha_{x} (1 - x) - \beta_{x} x = {{(x_{\infty } - x)} \mathord{\left/ {\vphantom {{(x_{\infty } - x)} {\tau_{x} }}} \right. \kern-0pt} {\tau_{x} }}$$

where $\alpha_{x}$ was the forward rate and $\beta_{x}$ was the backward rate. Table 1 provided the specific rate functions for *I*_Na_,* I*_K_.

**Table 1:** Rate functions.

Gating variable	Rate functions
*I*_Na_ activation(*a* = 3)	$\alpha { = }0.32{{(V_{m} + 50)} \mathord{\left/ {\vphantom {{(V_{m} + 50)} {(1 - \exp ({{ - (V_{m} + 50)} \mathord{\left/ {\vphantom {{ - (V_{m} + 50)} 4}} \right. \kern-0pt} 4}))}}} \right. \kern-0pt} {(1 - \exp ({{ - (V_{m} + 50)} \mathord{\left/ {\vphantom {{ - (V_{m} + 50)} 4}} \right. \kern-0pt} 4}))}}$
	$$\beta { = } - 0.28{{(V_{m} + 23)} \mathord{\left/ {\vphantom {{(V_{m} + 23)} {(1 - \exp ({{(V_{m} + 23)} \mathord{\left/ {\vphantom {{(V_{m} + 23)} 5}} \right. \kern-0pt} 5}))}}} \right. \kern-0pt} {(1 - \exp ({{(V_{m} + 23)} \mathord{\left/ {\vphantom {{(V_{m} + 23)} 5}} \right. \kern-0pt} 5}))}}$$
*I*_Na_ inactivation	$\alpha { = }0.128\exp ({{ - (V_{m} + 46)} \mathord{\left/ {\vphantom {{ - (V_{m} + 46)} {18}}} \right. \kern-0pt} {18}})$
	$$\beta { = }{4 \mathord{\left/ {\vphantom {4 {(1 + \exp ( - {{(V_{m} + 23)} \mathord{\left/ {\vphantom {{(V_{m} + 23)} 5}} \right. \kern-0pt} 5}))}}} \right. \kern-0pt} {(1 + \exp ( - {{(V_{m} + 23)} \mathord{\left/ {\vphantom {{(V_{m} + 23)} 5}} \right. \kern-0pt} 5}))}}$$
*I*_K_ activation(*a* = 4)	$\alpha { = }0.032{{(V_{m} + 48)} \mathord{\left/ {\vphantom {{(V_{m} + 48)} {(1 - \exp ({{ - (V_{m} + 48)} \mathord{\left/ {\vphantom {{ - (V_{m} + 48)} 5}} \right. \kern-0pt} 5}))}}} \right. \kern-0pt} {(1 - \exp ({{ - (V_{m} + 48)} \mathord{\left/ {\vphantom {{ - (V_{m} + 48)} 5}} \right. \kern-0pt} 5}))}}$
	$$\beta { = }0.5\exp ({{ - (V_{m} + 53)} \mathord{\left/ {\vphantom {{ - (V_{m} + 53)} {40}}} \right. \kern-0pt} {40}})$$

The low-threshold T-type Ca^2+^ current *I*_T_ was introduced in soma, proximal and distal dendrites, which is given by Eq. (3).

3 $$I_{T} = g_{T} m^{2} h\;(V_{m} - E_{T} )$$

where *E*_T_ = 120 mV, and *g*_T_ for soma, proximal and distal dendrites was 0.025 mS/cm^2^, 0.045 mS/cm^2^ and 0.6 mS/cm^2^ respectively. The kinetics of activation variable *m* and inactivation variable *h* were governed by Eq. (2). The expression for steady-state activation and inactivation functions was defined with Eq. (4).

4 $$\left\{ \begin{gathered} m_{\infty } = {1 \mathord{\left/ {\vphantom {1 {(1 + \exp ({{ - (V_{m} + 52)} \mathord{\left/ {\vphantom {{ - (V_{m} + 52)} {7.4}}} \right. \kern-0pt} {7.4}}))}}} \right. \kern-0pt} {(1 + \exp ({{ - (V_{m} + 52)} \mathord{\left/ {\vphantom {{ - (V_{m} + 52)} {7.4}}} \right. \kern-0pt} {7.4}}))}} \hfill \\ h_{\infty } = {1 \mathord{\left/ {\vphantom {1 {(1 + \exp ({{(V_{m} + 80)} \mathord{\left/ {\vphantom {{(V_{m} + 80)} 5}} \right. \kern-0pt} 5}))}}} \right. \kern-0pt} {(1 + \exp ({{(V_{m} + 80)} \mathord{\left/ {\vphantom {{(V_{m} + 80)} 5}} \right. \kern-0pt} 5}))}} \hfill \\ \end{gathered} \right.$$

And the voltage-dependent time constants for activation variable *m* and inactivation variable *h* were defined as follows:

5 $$\left\{ \begin{gathered} \tau_{m} = 1 + {{0.33} \mathord{\left/ {\vphantom {{0.33} {(\exp ({{(V_{m} + 27)} \mathord{\left/ {\vphantom {{(V_{m} + 27)} {10}}} \right. \kern-0pt} {10}}) + \exp ( - {{(V_{m} + 102)} \mathord{\left/ {\vphantom {{(V_{m} + 102)} {15}}} \right. \kern-0pt} {15}}))}}} \right. \kern-0pt} {(\exp ({{(V_{m} + 27)} \mathord{\left/ {\vphantom {{(V_{m} + 27)} {10}}} \right. \kern-0pt} {10}}) + \exp ( - {{(V_{m} + 102)} \mathord{\left/ {\vphantom {{(V_{m} + 102)} {15}}} \right. \kern-0pt} {15}}))}} \hfill \\ \tau_{h} = 28.3 + {{0.33} \mathord{\left/ {\vphantom {{0.33} {(\exp ({{(V_{m} + 48)} \mathord{\left/ {\vphantom {{(V_{m} + 48)} 4}} \right. \kern-0pt} 4}) + \exp ( - {{(V_{m} + 407)} \mathord{\left/ {\vphantom {{(V_{m} + 407)} {50}}} \right. \kern-0pt} {50}}))}}} \right. \kern-0pt} {(\exp ({{(V_{m} + 48)} \mathord{\left/ {\vphantom {{(V_{m} + 48)} 4}} \right. \kern-0pt} 4}) + \exp ( - {{(V_{m} + 407)} \mathord{\left/ {\vphantom {{(V_{m} + 407)} {50}}} \right. \kern-0pt} {50}}))}} \hfill \\ \end{gathered} \right.$$

To make the computational model more realistic, we modeled the calcium handling, including Ca^2+^ pumps and buffers, with by a first -order system with a time constant of decay of Ca^2+^ of 5 ms, which is shown as follow.

6 $$\frac{{d\left[ {Ca^{2 + } } \right]_{in} }}{dt} = - \frac{{10000I_{T} }}{2Fd} - \frac{{\left[ {Ca^{2 + } } \right]_{in} }}{{\left[ {Ca^{2 + } } \right]_{in} + K_{d} }} + \frac{{\left[ {Ca^{2 + } } \right]_{in} - \left[ {Ca^{2 + } } \right]_{res} }}{700}$$

where F = 96489C/mol represented Faraday constant, *d* = 0.1 μm^2^ represented the depth of shell under the cell membrane, *K*_d_ = 5 × 10^−4^ mM was the dissociation constant of the pump, and $\left[ {Ca^{2 + } } \right]_{res}$ = 240 nM was the calcium resting level. Here we focused on the variation of *I*_T_ rather than the dynamic of Ca^2+^ concentration. Unless otherwise stated, our stimulations were all based on this computational model which was implemented in NEURON with a constant time step of 0.1 ms. And the simulation ran for 3500 ms.

**Figure 5:**
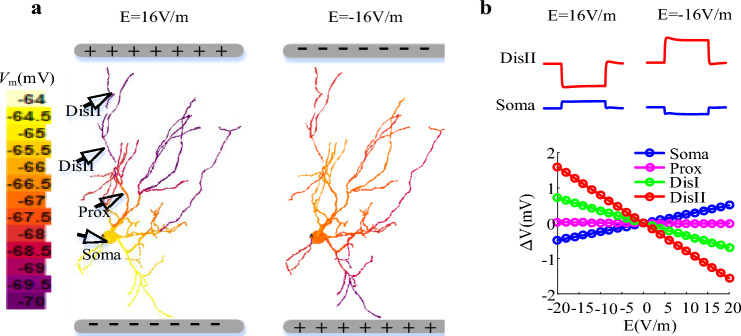
(**a**) False color map of membrane voltage *V*m at E = 16 V/m and − 16 V/m. (**b**) top panel: Voltage traces recorded in the soma and distal dendrite with two EFs. Bottom panel: membrane polarization $\Delta V_{m}$ in the soma (blue), proximal (magenta), medial (green) and distal (red) dendrites as EF ranges from − 20 V/m to 20 V/m. The recording sites are indicated by black triangles in (**a**).

The uniform EFs were applied to TRN neuron by using the extracellular mechanisms in NEURON, which has been described in Berzhanskaya et al.^35^ and Fan et al.^19^. As shown in Fig. 5, the EF stimulation with positive electrode loser to distal dendrites was referred to anodal stimulation (the left panel of Fig. 5a), which depolarized soma while hyperpolarizing distal dendrites (Fig. 5b). On the contrary, cathodal stimulation was referred to when the negative electrode was placed next to distal dendrites (the right panel of Fig. 5a), which could hyperpolarize soma while depolarizing distal dendrites (Fig. 5b). In our simulations, the EF ranged from − 100 V/m to 80 V/m. And E = 0 was referred to as the control case.

In vivo, both the soma/proximal dendrites and distal dendrites of TRN neuron receive synaptic inputs from other neurons. Here we first used a brief current pulse with a width of 7 ms to stimulate soma *I*_soma_ (in nA) or distal dendrite *I*_dend_, which allows us to observe the modulation effect of EF on neuronal output, such as tonic spike and burst discharge. And the smallest value of *I*_soma_ or *I*_dend_ for evoking AP was referred to the stimulation threshold. It is pointed out that the TRN neuron can produce state-independent dCa response^14^. Then to observe whether the state-independent dCa response depends on axial resistance, both pulse (30 ms;0.3 nA) and synaptic inputs were used to stimulate distal dendrites. The synaptic inputs included 100 AMPA (0.3 nS) synapses and 100 NMDA synapses(0.3 nS). The kinetics of the synaptic conductances were identical to the article of Poleg-Polsky^36^. AMPA currents had an instantaneous rise time of 0.2 ms and a decay time of 2 ms. NMDA currents had a rise time of 3 ms and a decay time of 70 ms. The conductance voltage dependence of NMDA synaptic inputs was modeled as follows: $g_{NMDA} = {1 \mathord{\left/ {\vphantom {1 {(1 + 0.3\exp ( - 0.08V_{m} ))}}} \right. \kern-0pt} {(1 + 0.3\exp ( - 0.08V_{m} ))}}$.

**Figure 6:**
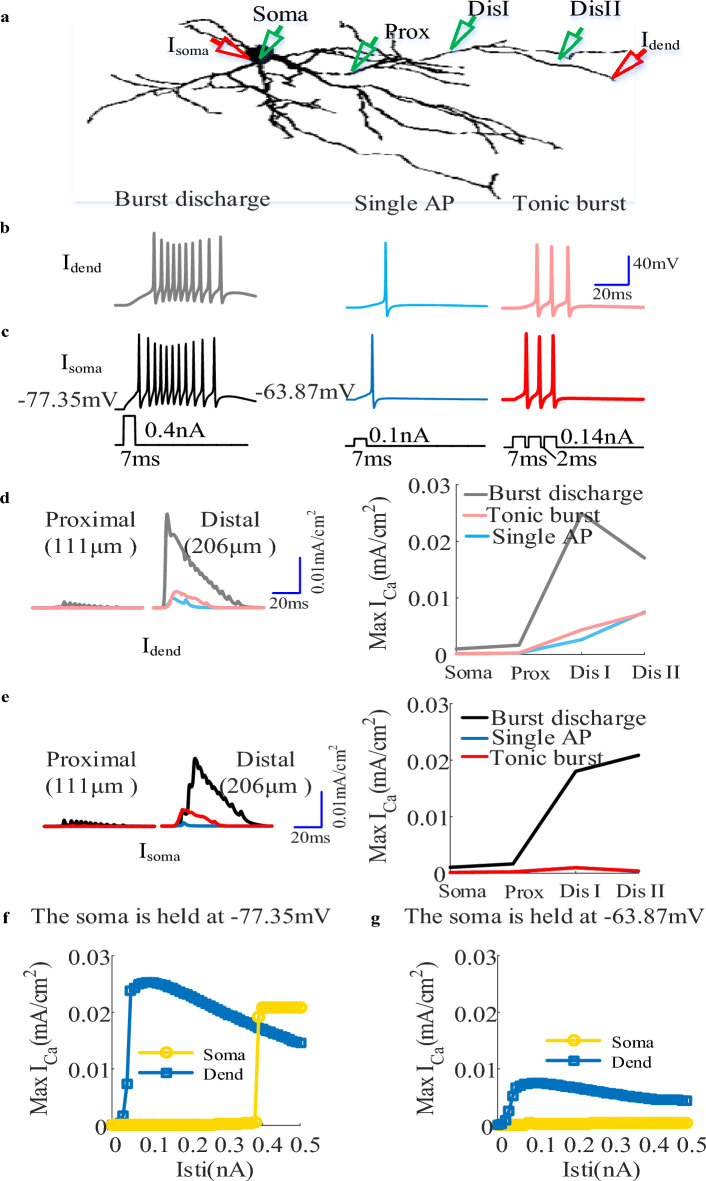
(**a**) The schematic of a TRN neuron to observe the impact of firing mode on Ca^2+^ response. The somatic and dendritic injection site was represented by red triangles, and the recording site at soma, proximal, medical and distal dendrites was represented the green triangles. (**b**) and (**c**) Action potentials were discharged in three ways. Burst discharge was elicited from a hyperpolarized holding potential (− 77.35 mV) by injecting a short depolarizing pulse at distal dendrite (grey) or at soma (black). A single action potential was evoked by holding the soma near − 63.87 mV and injecting short depolarizing current pulses at distal dendrite (light blue) and soma (dark blue). Similarly, at − 63.87 mV a tonic burst of action potentials was triggered by periodic stimulation at soma (red) and distal dendrite (light red). (**d**) Left: the variation of *I*_T_ with time at proximal and distal dendrites as the TRN neuron   was discharged in three ways that was shown in (**b**). Right: the peak Ca^2+^ response at soma, proximal, medical and distal dendrites in case of burst discharge (grey), tonic burst (light blue), and single action potentials (light red). (**e**) similar to (**d**) but for somatic input. (**f**) and (**g**) The peak Ca^2+^ response recorded at the distal dendrite for increasing dendritic (blue) and somatic (yellow) current pulse when the initial membrane potential of soma was held at − 77.35 mV and − 63.87 mV respectively. Here, E = 0.

As described in Introduction, the voltage state affects the output of TRN cell by determining the existence of dCa response^14^, which can be used to validate the effectiveness of the computational model. Thus, we applied current injection at soma and distal dendrites and observed the transmembrane potentials in the soma and the dCa response along the entire dendrites of TRN model, as shown in Fig. 6. Here, the dCa response was obtained by computing the peak value of *I*_T_. We found that at relatively hyperpolarized state (− 77.35 mV) TRN neuron generates burst discharge in response to current injection via activating *I*_T_ (Fig. 6b,c). In this case, there existed more pronounced Ca^2+^ response at distal dendrites than that at proximal dendrites (Fig. 6d,e). Differently, as the TRN cell receives somatic and dendritic inputs at relatively depolarized state(− 63.87 mV) we observed a single AP evoked by a brief current pulse and a tonic burst of AP evoked by a periodic stimulation (Fig. 6b,c). Under this condition, there exists small Ca^2+^ response at distal dendrites due to the inactivation of *I*_T_ (Fig. 6d,e). These results suggest that an underlying LTS must be responsible for the large dCa response, which is consistent with the experimental finding of Crandall et al.^14^. Then we characterized the threshold for triggering LTS at relatively hyperpolarized and depolarized state. At − 63.87 mV both dendritic and somatic inputs only evoke weak dCa response (Fig. 6g). At − 77.35 mV the threshold for evoking LTS is 0.019 mA/cm^2^ as the neuron receives somatic inputs while for dendritic inputs the threshold is 0.021 mA/cm^2^ (Fig. 6f). Thus we defined I_Ca_thre_ = 0.019 mA/cm^2^ as the threshold for triggering large dCa response.

## Data Availability

All data generated or analysed during this study are included in this article.

## Code availability

Custom code is available upon reasonable request.
